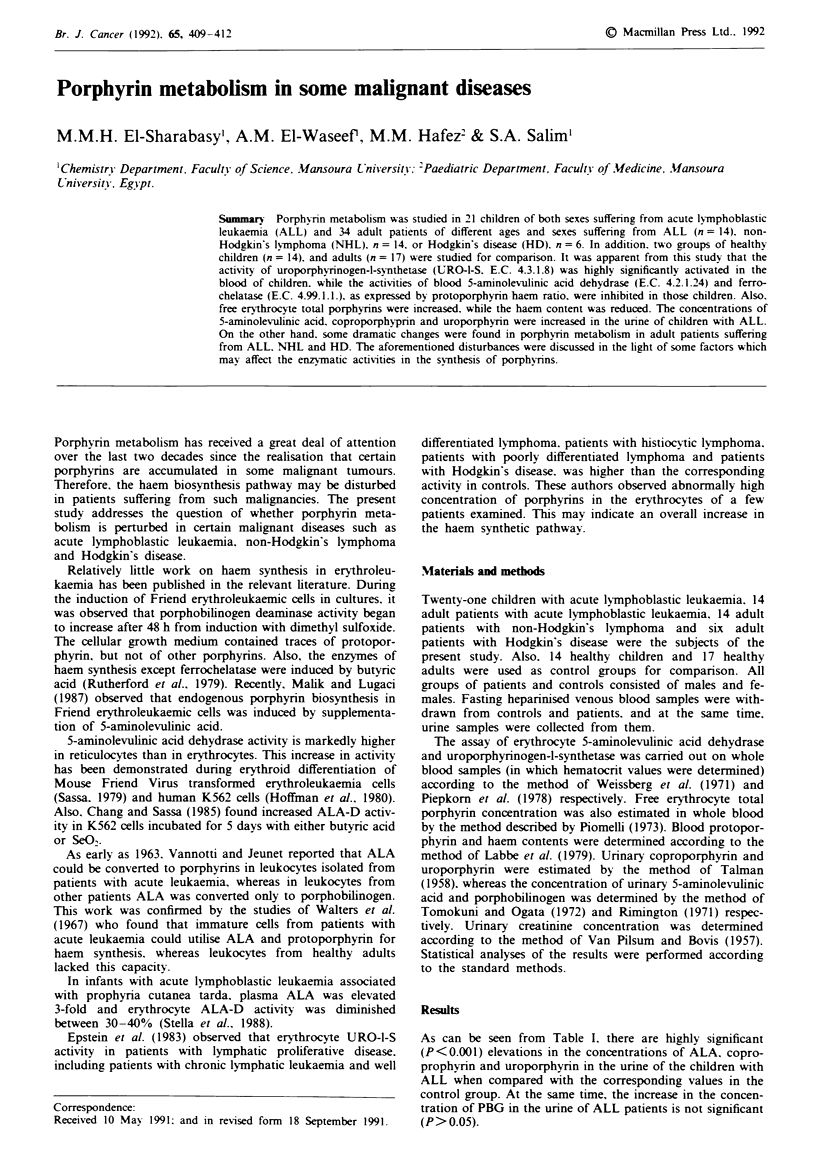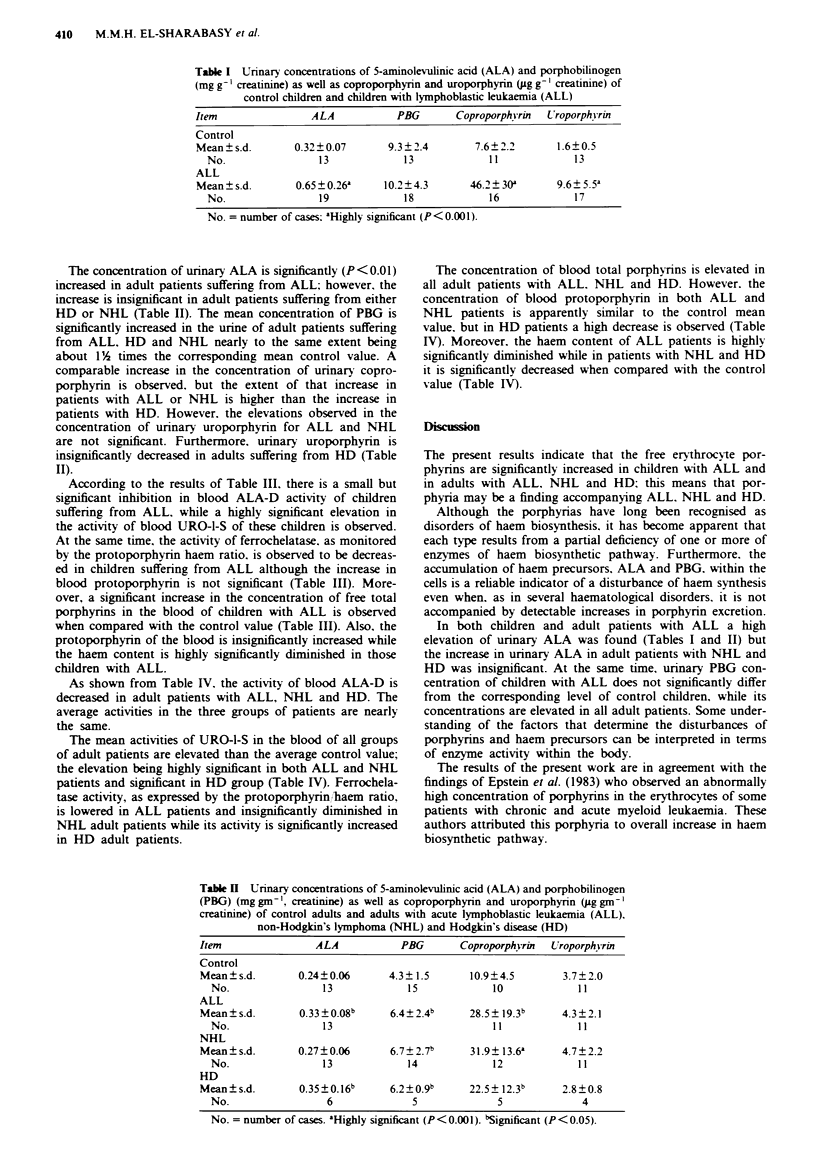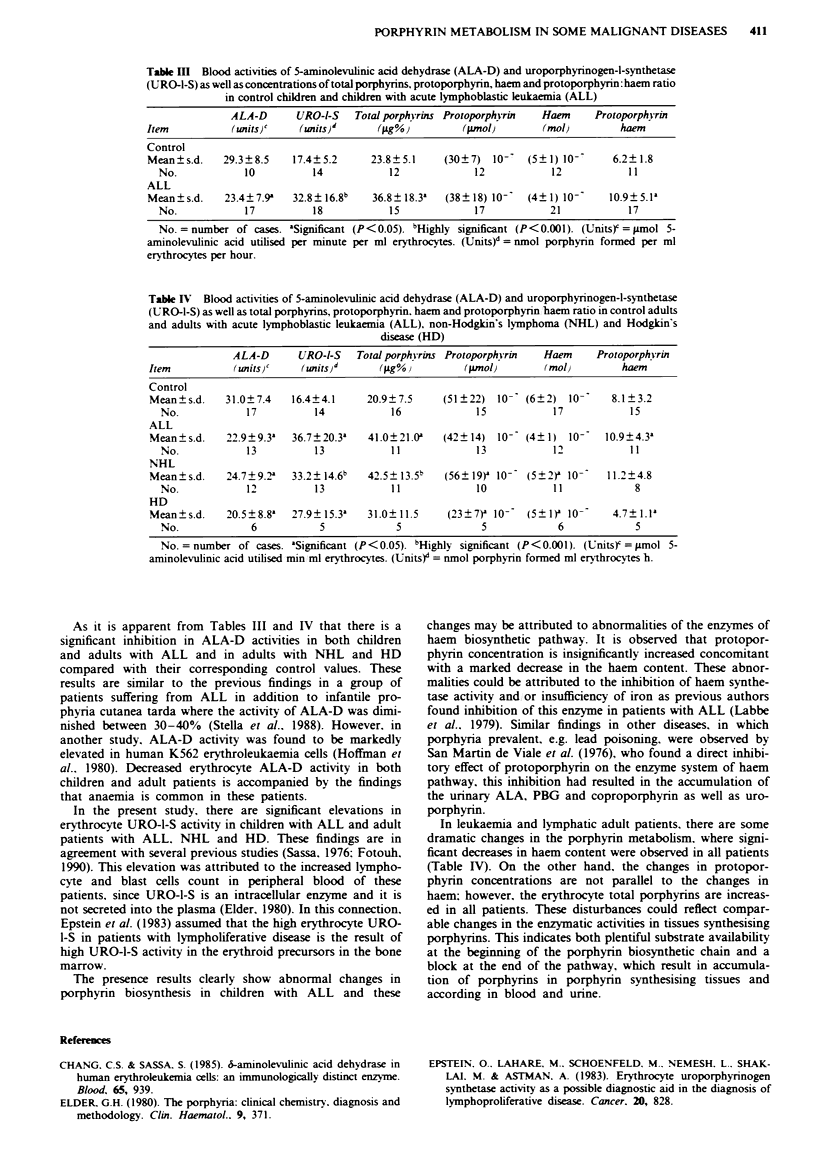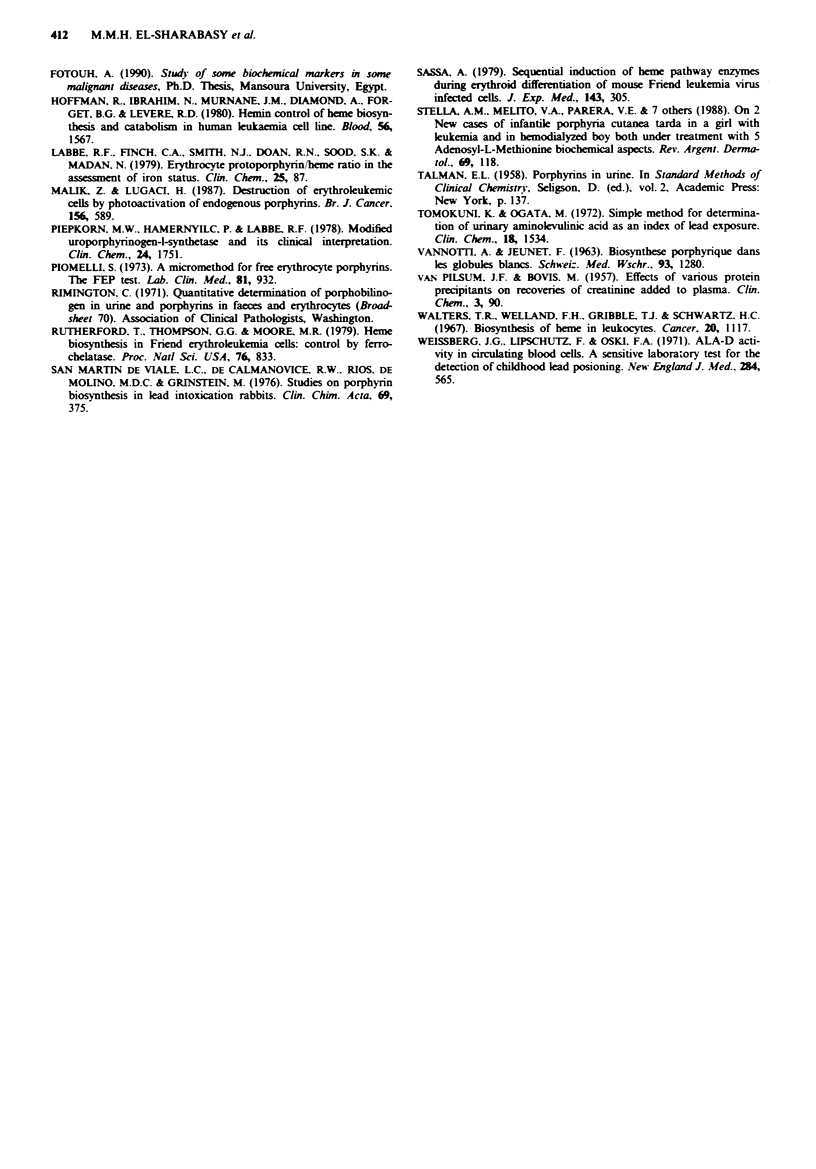# Porphyrin metabolism in some malignant diseases.

**DOI:** 10.1038/bjc.1992.83

**Published:** 1992-03

**Authors:** M. M. el-Sharabasy, A. M. el-Waseef, M. M. Hafez, S. A. Salim

**Affiliations:** Chemistry Department, Faculty of Science, Mansoura University, Egypt.

## Abstract

Porphyrin metabolism was studied in 21 children of both sexes suffering from acute lymphoblastic leukaemia (ALL) and 34 adult patients of different ages and sexes suffering from ALL (n = 14), non-Hodgkin's lymphoma (NHL), n = 14, or Hodgkin's disease (HD), n = 6. In addition, two groups of healthy children (n = 14), and adults (n = 17) were studied for comparison. It was apparent from this study that the activity of uroporphyrinogen-1-synthetase (URO-1-S, E.C. 4.3.1.8) was highly significantly activated in the blood of children, while the activities of blood 5-aminolevulinic acid dehydrase (E.C. 4.2.1.24) and ferrochelatase (E.C. 4.99.1.1.), as expressed by protoporphyrin/haem ratio, were inhibited in those children. Also, free erythrocyte total porphyrins were increased, while the haem content was reduced. The concentrations of 5-aminolevulinic acid, coproporphyprin and uroporphyrin were increased in the urine of children with ALL. On the other hand, some dramatic changes were found in porphyrin metabolism in adult patients suffering from ALL, NHL and HD. The aforementioned disturbances were discussed in the light of some factors which may affect the enzymatic activities in the synthesis of porphyrins.


 © Macmillan Press Ltd., 1992

### Porphyrin metabolism in some malignant diseases

M.M.H. El-Sharabasy[1], A.M. El-Waseef[1], M.M. Hafez[2] & S.A. Salim[1]

[1]*Chemistry Department, Faculty of Science, Mansoura University;* [2]*Paediatric Department, Faculty of Medicine, Mansoura University, Egypt.*

**Summary** Porphyrin metabolism was studied in 21 children of both sexes suffering from acute lymphoblastic leukaemia (ALL) and 34 adult patients of different ages and sexes suffering from ALL ($n = 14$), non-Hodgkin's lymphoma (NHL), $n = 14$, or Hodgkin's disease (HD), $n = 6$. In addition, two groups of healthy children ($n = 14$), and adults ($n = 17$) were studied for comparison. It was apparent from this study that the activity of uroporphyrinogen-I-synthetase (URO-I-S, E.C. 4.3.1.8) was highly significantly activated in the blood of children, while the activities of blood 5-aminolevulinic acid dehydrase (E.C. 4.2.1.24) and ferrochelatase (E.C. 4.99.1.1.), as expressed by protoporphyrin haem ratio, were inhibited in those children. Also, free erythrocyte total porphyrins were increased, while the haem content was reduced. The concentrations of 5-aminolevulinic acid, coproporphyprin and uroporphyrin were increased in the urine of children with ALL. On the other hand, some dramatic changes were found in porphyrin metabolism in adult patients suffering from ALL, NHL and HD. The aforementioned disturbances were discussed in the light of some factors which may affect the enzymatic activities in the synthesis of porphyrins.

Porphyrin metabolism has received a great deal of attention over the last two decades since the realisation that certain porphyrins are accumulated in some malignant tumours. Therefore, the haem biosynthesis pathway may be disturbed in patients suffering from such malignancies. The present study addresses the question of whether porphyrin metabolism is perturbed in certain malignant diseases such as acute lymphoblastic leukaemia, non-Hodgkin's lymphoma and Hodgkin's disease.

Relatively little work on haem synthesis in erythroleukaemia has been published in the relevant literature. During the induction of Friend erythroleukaemic cells in cultures, it was observed that porphobilinogen deaminase activity began to increase after 48 h from induction with dimethyl sulfoxide. The cellular growth medium contained traces of protoporphyrin, but not of other porphyrins. Also, the enzymes of haem synthesis except ferrochelatase were induced by butyric acid (Rutherford *et al.*, 1979). Recently, Malik and Lugaci (1987) observed that endogenous porphyrin biosynthesis in Friend erythroleukaemic cells was induced by supplementation of 5-aminolevulinic acid.

5-aminolevulinic acid dehydrase activity is markedly higher in reticulocytes than in erythrocytes. This increase in activity has been demonstrated during erythroid differentiation of Mouse Friend Virus transformed erythroleukaemia cells (Sassa, 1979) and human K562 cells (Hoffman *et al.*, 1980). Also, Chang and Sassa (1985) found increased ALA-D activity in K562 cells incubated for 5 days with either butyric acid or $SeO_2$.

As early as 1963, Vannotti and Jeunet reported that ALA could be converted to porphyrins in leukocytes isolated from patients with acute leukaemia, whereas in leukocytes from other patients ALA was converted only to porphobilinogen. This work was confirmed by the studies of Walters *et al.* (1967) who found that immature cells from patients with acute leukaemia could utilise ALA and protoporphyrin for haem synthesis, whereas leukocytes from healthy adults lacked this capacity.

In infants with acute lymphoblastic leukaemia associated with prophyria cutanea tarda, plasma ALA was elevated 3-fold and erythrocyte ALA-D activity was diminished between 30–40% (Stella *et al.*, 1988).

Epstein *et al.* (1983) observed that erythrocyte URO-I-S activity in patients with lymphatic proliferative disease, including patients with chronic lymphatic leukaemia and well differentiated lymphoma, patients with histiocytic lymphoma, patients with poorly differentiated lymphoma and patients with Hodgkin's disease, was higher than the corresponding activity in controls. These authors observed abnormally high concentration of porphyrins in the erythrocytes of a few patients examined. This may indicate an overall increase in the haem synthetic pathway.

### Materials and methods

Twenty-one children with acute lymphoblastic leukaemia, 14 adult patients with acute lymphoblastic leukaemia, 14 adult patients with non-Hodgkin's lymphoma and six adult patients with Hodgkin's disease were the subjects of the present study. Also, 14 healthy children and 17 healthy adults were used as control groups for comparison. All groups of patients and controls consisted of males and females. Fasting heparinised venous blood samples were withdrawn from controls and patients, and at the same time, urine samples were collected from them.

The assay of erythrocyte 5-aminolevulinic acid dehydrase and uroporphyrinogen-I-synthetase was carried out on whole blood samples (in which hematocrit values were determined) according to the method of Weissberg *et al.* (1971) and Piepkorn *et al.* (1978) respectively. Free erythrocyte total porphyrin concentration was also estimated in whole blood by the method described by Piomelli (1973). Blood protoporphyrin and haem contents were determined according to the method of Labbe *et al.* (1979). Urinary coproporphyrin and uroporphyrin were estimated by the method of Talman (1958), whereas the concentration of urinary 5-aminolevulinic acid and porphobilinogen was determined by the method of Tomokuni and Ogata (1972) and Rimington (1971) respectively. Urinary creatinine concentration was determined according to the method of Van Pilsum and Bovis (1957). Statistical analyses of the results were performed according to the standard methods.

### Results

As can be seen from Table I, there are highly significant ($P < 0.001$) elevations in the concentrations of ALA, coproprophyrin and uroporphyrin in the urine of the children with ALL when compared with the corresponding values in the control group. At the same time, the increase in the concentration of PBG in the urine of ALL patients is not significant ($P > 0.05$).

Correspondence:
Received 10 May 1991; and in revised form 18 September 1991.

**Table I** Urinary concentrations of 5-aminolevulinic acid (ALA) and porphobilinogen (mg $g^{-1}$ creatinine) as well as coproporphyrin and uroporphyrin (μg $g^{-1}$ creatinine) of control children and children with lymphoblastic leukaemia (ALL)

| *Item* | *ALA* | *PBG* | *Coproporphyrin* | *Uroporphyrin* |
|---|---|---|---|---|
| Control | | | | |
| Mean ± s.d. | 0.32 ± 0.07 | 9.3 ± 2.4 | 7.6 ± 2.2 | 1.6 ± 0.5 |
| No. | 13 | 13 | 11 | 13 |
| ALL | | | | |
| Mean ± s.d. | 0.65 ± 0.26[a] | 10.2 ± 4.3 | 46.2 ± 30[a] | 9.6 ± 5.5[a] |
| No. | 19 | 18 | 16 | 17 |

No. = number of cases; [a]Highly significant ($P < 0.001$).

The concentration of urinary ALA is significantly ($P < 0.01$) increased in adult patients suffering from ALL; however, the increase is insignificant in adult patients suffering from either HD or NHL (Table II). The mean concentration of PBG is significantly increased in the urine of adult patients suffering from ALL, HD and NHL nearly to the same extent being about 1½ times the corresponding mean control value. A comparable increase in the concentration of urinary coproporphyrin is observed, but the extent of that increase in patients with ALL or NHL is higher than the increase in patients with HD. However, the elevations observed in the concentration of urinary uroporphyrin for ALL and NHL are not significant. Furthermore, urinary uroporphyrin is insignificantly decreased in adults suffering from HD (Table II).

According to the results of Table III, there is a small but significant inhibition in blood ALA-D activity of children suffering from ALL, while a highly significant elevation in the activity of blood URO-I-S of these children is observed. At the same time, the activity of ferrochelatase, as monitored by the protoporphyrin/haem ratio, is observed to be decreased in children suffering from ALL although the increase in blood protoporphyrin is not significant (Table III). Moreover, a significant increase in the concentration of free total porphyrins in the blood of children with ALL is observed when compared with the control value (Table III). Also, the protoporphyrin of the blood is insignificantly increased while the haem content is highly significantly diminished in those children with ALL.

As shown from Table IV, the activity of blood ALA-D is decreased in adult patients with ALL, NHL and HD. The average activities in the three groups of patients are nearly the same.

The mean activities of URO-I-S in the blood of all groups of adult patients are elevated than the average control value; the elevation being highly significant in both ALL and NHL patients and significant in HD group (Table IV). Ferrochelatase activity, as expressed by the protoporphyrin/haem ratio, is lowered in ALL patients and insignificantly diminished in NHL adult patients while its activity is significantly increased in HD adult patients.

The concentration of blood total porphyrins is elevated in all adult patients with ALL, NHL and HD. However, the concentration of blood protoporphyrin in both ALL and NHL patients is apparently similar to the control mean value, but in HD patients a high decrease is observed (Table IV). Moreover, the haem content of ALL patients is highly significantly diminished while in patients with NHL and HD it is significantly decreased when compared with the control value (Table IV).

### Discussion

The present results indicate that the free erythrocyte porphyrins are significantly increased in children with ALL and in adults with ALL, NHL and HD; this means that porphyria may be a finding accompanying ALL, NHL and HD.

Although the porphyrias have long been recognised as disorders of haem biosynthesis, it has become apparent that each type results from a partial deficiency of one or more of enzymes of haem biosynthetic pathway. Furthermore, the accumulation of haem precursors, ALA and PBG, within the cells is a reliable indicator of a disturbance of haem synthesis even when, as in several haematological disorders, it is not accompanied by detectable increases in porphyrin excretion.

In both children and adult patients with ALL a high elevation of urinary ALA was found (Tables I and II) but the increase in urinary ALA in adult patients with NHL and HD was insignificant. At the same time, urinary PBG concentration of children with ALL does not significantly differ from the corresponding level of control children, while its concentrations are elevated in all adult patients. Some understanding of the factors that determine the disturbances of porphyrins and haem precursors can be interpreted in terms of enzyme activity within the body.

The results of the present work are in agreement with the findings of Epstein *et al.* (1983) who observed an abnormally high concentration of porphyrins in the erythrocytes of some patients with chronic and acute myeloid leukaemia. These authors attributed this porphyria to overall increase in haem biosynthetic pathway.

**Table II** Urinary concentrations of 5-aminolevulinic acid (ALA) and porphobilinogen (PBG) (mg $gm^{-1}$, creatinine) as well as coproporphyrin and uroporphyrin (μg $gm^{-1}$ creatinine) of control adults and adults with acute lymphoblastic leukaemia (ALL), non-Hodgkin's lymphoma (NHL) and Hodgkin's disease (HD)

| *Item* | *ALA* | *PBG* | *Coproporphyrin* | *Uroporphyrin* |
|---|---|---|---|---|
| Control | | | | |
| Mean ± s.d. | 0.24 ± 0.06 | 4.3 ± 1.5 | 10.9 ± 4.5 | 3.7 ± 2.0 |
| No. | 13 | 15 | 10 | 11 |
| ALL | | | | |
| Mean ± s.d. | 0.33 ± 0.08[b] | 6.4 ± 2.4[b] | 28.5 ± 19.3[b] | 4.3 ± 2.1 |
| No. | 13 | | 11 | 11 |
| NHL | | | | |
| Mean ± s.d. | 0.27 ± 0.06 | 6.7 ± 2.7[b] | 31.9 ± 13.6[a] | 4.7 ± 2.2 |
| No. | 13 | 14 | 12 | 11 |
| HD | | | | |
| Mean ± s.d. | 0.35 ± 0.16[b] | 6.2 ± 0.9[b] | 22.5 ± 12.3[b] | 2.8 ± 0.8 |
| No. | 6 | 5 | 5 | 4 |

No. = number of cases. [a]Highly significant ($P < 0.001$). [b]Significant ($P < 0.05$).

**Table III** Blood activities of 5-aminolevulinic acid dehydrase (ALA-D) and uroporphyrinogen-l-synthetase (URO-l-S) as well as concentrations of total porphyrins, protoporphyrin, haem and protoporphyrin:haem ratio in control children and children with acute lymphoblastic leukaemia (ALL)

| *Item* | *ALA-D (units)*[c] | *URO-l-S (units)*[d] | *Total porphyrins (μg%)* | *Protoporphyrin (μmol)* | *Haem (mol)* | *Protoporphyrin haem* |
|---|---|---|---|---|---|---|
| Control | | | | | | |
| Mean ± s.d. | 29.3±8.5 | 17.4±5.2 | 23.8±5.1 | (30±7) $10^{-7}$ | (5±1) $10^{-7}$ | 6.2±1.8 |
| No. | 10 | 14 | 12 | 12 | 12 | 11 |
| ALL | | | | | | |
| Mean ± s.d. | 23.4±7.9[a] | 32.8±16.8[b] | 36.8±18.3[a] | (38±18) $10^{-7}$ | (4±1) $10^{-7}$ | 10.9±5.1[a] |
| No. | 17 | 18 | 15 | 17 | 21 | 17 |

No. = number of cases. [a]Significant ($P<0.05$). [b]Highly significant ($P<0.001$). (Units)[c] = μmol 5-aminolevulinic acid utilised per minute per ml erythrocytes. (Units)[d] = nmol porphyrin formed per ml erythrocytes per hour.

**Table IV** Blood activities of 5-aminolevulinic acid dehydrase (ALA-D) and uroporphyrinogen-l-synthetase (URO-l-S) as well as total porphyrins, protoporphyrin, haem and protoporphyrin/haem ratio in control adults and adults with acute lymphoblastic leukaemia (ALL), non-Hodgkin's lymphoma (NHL) and Hodgkin's disease (HD)

| *Item* | *ALA-D (units)*[c] | *URO-l-S (units)*[d] | *Total porphyrins (μg%)* | *Protoporphyrin (μmol)* | *Haem (mol)* | *Protoporphyrin haem* |
|---|---|---|---|---|---|---|
| Control | | | | | | |
| Mean ± s.d. | 31.0±7.4 | 16.4±4.1 | 20.9±7.5 | (51±22) $10^{-7}$ | (6±2) $10^{-7}$ | 8.1±3.2 |
| No. | 17 | 14 | 16 | 15 | 17 | 15 |
| ALL | | | | | | |
| Mean ± s.d. | 22.9±9.3[a] | 36.7±20.3[a] | 41.0±21.0[a] | (42±14) $10^{-7}$ | (4±1) $10^{-7}$ | 10.9±4.3[a] |
| No. | 13 | 13 | 11 | 13 | 12 | 11 |
| NHL | | | | | | |
| Mean ± s.d. | 24.7±9.2[a] | 33.2±14.6[b] | 42.5±13.5[b] | (56±19)[a] $10^{-7}$ | (5±2)[a] $10^{-7}$ | 11.2±4.8 |
| No. | 12 | 13 | 11 | 10 | 11 | 8 |
| HD | | | | | | |
| Mean ± s.d. | 20.5±8.8[a] | 27.9±15.3[a] | 31.0±11.5 | (23±7)[a] $10^{-7}$ | (5±1)[a] $10^{-7}$ | 4.7±1.1[a] |
| No. | 6 | 5 | 5 | 5 | 6 | 5 |

No. = number of cases. [a]Significant ($P<0.05$). [b]Highly significant ($P<0.001$). (Units)[c] = μmol 5-aminolevulinic acid utilised min/ml erythrocytes. (Units)[d] = nmol porphyrin formed ml erythrocytes h.

As it is apparent from Tables III and IV that there is a significant inhibition in ALA-D activities in both children and adults with ALL and in adults with NHL and HD compared with their corresponding control values. These results are similar to the previous findings in a group of patients suffering from ALL in addition to infantile prophyria cutanea tarda where the activity of ALA-D was diminished between 30–40% (Stella *et al.*, 1988). However, in another study, ALA-D activity was found to be markedly elevated in human K562 erythroleukaemia cells (Hoffman *et al.*, 1980). Decreased erythrocyte ALA-D activity in both children and adult patients is accompanied by the findings that anaemia is common in these patients.

In the present study, there are significant elevations in erythrocyte URO-l-S activity in children with ALL and adult patients with ALL, NHL and HD. These findings are in agreement with several previous studies (Sassa, 1976; Fotouh, 1990). This elevation was attributed to the increased lymphocyte and blast cells count in peripheral blood of these patients, since URO-l-S is an intracellular enzyme and it is not secreted into the plasma (Elder, 1980). In this connection, Epstein *et al.* (1983) assumed that the high erythrocyte URO-l-S in patients with lympholiferative disease is the result of high URO-l-S activity in the erythroid precursors in the bone marrow.

The presence results clearly show abnormal changes in porphyrin biosynthesis in children with ALL and these changes may be attributed to abnormalities of the enzymes of haem biosynthetic pathway. It is observed that protoporphyrin concentration is insignificantly increased concomitant with a marked decrease in the haem content. These abnormalities could be attributed to the inhibition of haem synthetase activity and/or insufficiency of iron as previous authors found inhibition of this enzyme in patients with ALL (Labbe *et al.*, 1979). Similar findings in other diseases, in which porphyria prevalent, e.g. lead poisoning, were observed by San Martin de Viale *et al.* (1976), who found a direct inhibitory effect of protoporphyrin on the enzyme system of haem pathway, this inhibition had resulted in the accumulation of the urinary ALA, PBG and coproporphyrin as well as uroporphyrin.

In leukaemia and lymphatic adult patients, there are some dramatic changes in the porphyrin metabolism, where significant decreases in haem content were observed in all patients (Table IV). On the other hand, the changes in protoporphyrin concentrations are not parallel to the changes in haem; however, the erythrocyte total porphyrins are increased in all patients. These disturbances could reflect comparable changes in the enzymatic activities in tissues synthesising porphyrins. This indicates both plentiful substrate availability at the beginning of the porphyrin biosynthetic chain and a block at the end of the pathway, which result in accumulation of porphyrins in porphyrin synthesising tissues and according in blood and urine.

### References

CHANG, C.S. & SASSA, S. (1985). δ-aminolevulinic acid dehydrase in human erythroleukemia cells: an immunologically distinct enzyme. *Blood*, **65**, 939.

ELDER, G.H. (1980). The porphyria: clinical chemistry, diagnosis and methodology. *Clin. Haematol.*, **9**, 371.

EPSTEIN, O., LAHARE, M., SCHOENFELD, M., NEMESH, L., SHAKLAI, M. & ASTMAN, A. (1983). Erythrocyte uroporphyrinogen synthetase activity as a possible diagnostic aid in the diagnosis of lymphoproliferative disease. *Cancer*, **20**, 828.

FOTOUH, A. (1990). *Study of some biochemical markers in some malignant diseases*, Ph.D. Thesis, Mansoura University, Egypt.

HOFFMAN, R., IBRAHIM, N., MURNANE, J.M., DIAMOND, A., FORGET, B.G. & LEVERE, R.D. (1980). Hemin control of heme biosynthesis and catabolism in human leukaemia cell line. *Blood*, **56**, 1567.

LABBE, R.F., FINCH, C.A., SMITH, N.J., DOAN, R.N., SOOD, S.K. & MADAN, N. (1979). Erythrocyte protoporphyrin/heme ratio in the assessment of iron status. *Clin. Chem.*, **25**, 87.

MALIK, Z. & LUGACI, H. (1987). Destruction of erythroleukemic cells by photoactivation of endogenous porphyrins. *Br. J. Cancer*, **156**, 589.

PIEPKORN, M.W., HAMERNYILC, P. & LABBE, R.F. (1978). Modified uroporphyrinogen-l-synthetase and its clinical interpretation. *Clin. Chem.*, **24**, 1751.

PIOMELLI, S. (1973). A micromethod for free erythrocyte porphyrins. The FEP test. *Lab. Clin. Med.*, **81**, 932.

RIMINGTON, C. (1971). Quantitative determination of porphobilinogen in urine and porphyrins in faeces and erythrocytes (*Broadsheet* 70). Association of Clinical Pathologists, Washington.

RUTHERFORD, T., THOMPSON, G.G. & MOORE, M.R. (1979). Heme biosynthesis in Friend erythroleukemia cells: control by ferrochelatase. *Proc. Natl Sci. USA*, **76**, 833.

SAN MARTIN DE VIALE, L.C., DE CALMANOVICE, R.W., RIOS, DE MOLINO, M.D.C. & GRINSTEIN, M. (1976). Studies on porphyrin biosynthesis in lead intoxication rabbits. *Clin. Chim. Acta*, **69**, 375.

SASSA, A. (1979). Sequential induction of heme pathway enzymes during erythroid differentiation of mouse Friend leukemia virus infected cells. *J. Exp. Med.*, **143**, 305.

STELLA, A.M., MELITO, V.A., PARERA, V.E. & 7 others (1988). On 2 New cases of infantile porphyria cutanea tarda in a girl with leukemia and in hemodialyzed boy both under treatment with 5 Adenosyl-L-Methionine biochemical aspects. *Rev. Argent. Dermatol.*, **69**, 118.

TALMAN, E.L. (1958). Porphyrins in urine. In *Standard Methods of Clinical Chemistry*, Seligson, D. (ed.), vol. 2, Academic Press: New York, p. 137.

TOMOKUNI, K. & OGATA, M. (1972). Simple method for determination of urinary aminolevulinic acid as an index of lead exposure. *Clin. Chem.*, **18**, 1534.

VANNOTTI, A. & JEUNET, F. (1963). Biosynthese porphyrique dans les globules blancs. *Schweiz. Med. Wschr.*, **93**, 1280.

VAN PILSUM, J.F. & BOVIS, M. (1957). Effects of various protein precipitants on recoveries of creatinine added to plasma. *Clin. Chem.*, **3**, 90.

WALTERS, T.R., WELLAND, F.H., GRIBBLE, T.J. & SCHWARTZ, H.C. (1967). Biosynthesis of heme in leukocytes. *Cancer*, **20**, 1117.

WEISSBERG, J.G., LIPSCHUTZ, F. & OSKI, F.A. (1971). ALA-D activity in circulating blood cells. A sensitive laboratory test for the detection of childhood lead posioning. *New England J. Med.*, **284**, 565.